# “They’re Lacking Purpose. It’s a Recipe for Suicide.”: Masculinity and Gender-Based Inequalities in Deaths of Despair in England

**DOI:** 10.1177/15579883251329715

**Published:** 2025-05-01

**Authors:** Timothy Price

**Affiliations:** 1 Population Health Sciences Institute, Faculty of Medical Sciences, Newcastle University, Newcastle upon Tyne, UK

**Keywords:** deaths of despair, suicide, behavioural issues, social determinants of health, psychosocial and cultural issues, masculinity, gender issues and sexual orientation

## Abstract

This qualitative study explores the factors contributing to gender-based inequalities in “deaths of despair” (DoD) – those deaths from suicide, drug overdoses, and alcohol-specific causes – among men in two deindustrialised towns in North East England. Understanding gender-based disparities in these causes of death sheds important light on how social and economic factors intersect with certain facets of masculinity, such as stoicism and self-reliance, to drive vulnerability. Data were collected through semi-structured interviews and one focus group with 54 stakeholders – people whose work is related to DoD, such as mental health treatment and public health practice – and community members in Middlesbrough and South Tyneside, two towns with above average rates of DoD. Participants included both men and women, predominantly of middle-age or older, with most participants unemployed or retired. Data were analysed using Iterative Categorisation, with findings interpreted through thematic analysis. The study generated three key themes: industrial history and masculinity, masculinity and class, and masculinity as a barrier to help-seeking. The findings demonstrate that economic decline following deindustrialisation resulted in distress and created unique risk factors for substance use and self-harm for men. These results suggest that effective interventions to reduce DoD among men in post-industrial settings must avoid pathologising masculinity itself. Instead, strategies should focus on the broader structural forces that undermine men’s access to stable, fulfilling employment and offering forms of support that are compatible with masculine identity. By addressing these determinants, interventions can more effectively close gender-based inequalities and reduce the rate of DoD in deindustrialised areas.

## Introduction

In 2015, economists Anne Case and Angus Deaton identified that growing mortality due to drugs, suicide, and alcohol-specific causes among middle-aged non-Hispanic White men without a college education had caused a stagnation in overall life expectancy in the United States (Case & Deaton, 2015). Case and Deaton labelled deaths from these causes “deaths of despair” (DoD) and proposed that they were driven by cumulative economic disadvantage (e.g. wage stagnation, low economic mobility) and the breakdown of traditional social structures and institutions (e.g. age at marriage, religious participation) (Case and Deaton, 2017, 2020). Much of the research on DoD has been conducted in the United States, where the increase in DoD since the 1990s has been most dramatic (Beseran et al., 2022; Cataldo, 2022; DeVerteuil, 2022; Friedman & Hansen, 2024; Gold, 2020; E. Harris, 2024); however, there is growing interest in DoD among researchers in other countries (Bastiampillai et al., 2021; Loverock et al., 2024; Piñeiro et al., 2023). The available evidence in both the United States and United Kingdom suggests that DoD are closely tied to economic, political, and social factors (Bjorklund, 2023; Dowd et al., 2023; Knapp et al., 2019; Price et al., 2024).

While the increase in DoD mortality rates in the United Kingdom has been less significant than those in the United States, epidemiological evidence shows that rates of DoD in England and Wales specifically increased by 21.6% in men and 16.9% in women between 2001 and 2016 (Augarde et al., 2022). Increases in rates of DoD in this context have been driven primarily by increases in drug-related mortality, with alcohol-specific mortality and deaths by suicide increasing at a more moderate rate (Augarde et al., 2022; Dowd et al., 2023), a pattern that mirrors the rise of DoD in the United States (Ruhm, 2021). Within the United Kingdom, geographic inequalities in DoD persist between constituent countries, with Scotland experiencing the greatest increases in rates of DoD (Allik et al., 2020; Walsh et al., 2021). Within England, geographic inequalities persist between regions, with people in the North East bearing a greater burden of DoD than their southern counterparts, and between cities within regions (Camacho et al., 2024). That the north of England is home to above national-average rates of DoD is consistent with the evidence surrounding regional health inequalities more broadly, a phenomenon known as the North-South divide in health (Bambra, 2016; Bernard et al., 2024). The North of England has a history of significant deindustrialisation at the end of the 20th century (Hudson, 1986; Telford & Lloyd, 2020), a factor that has been associated with increased rates of DoD in the United States and Eastern Europe (Herzog, 2020; King et al., 2022; Steelesmith et al., 2023; Stuckler et al., 2009).

DoD disproportionately affect men. Indeed, Case and Deaton’s 2017 paper, in which Case and Deaton first used the term “DoD” noted that the decrease in overall life expectancy in the United States was attributable to increased midlife mortality for non-Hispanic White *men* specifically (Case and Deaton, 2015, 2017). The gender disparity in DoD is not unique to the United States; globally, men experience DoD at a 3.3-fold higher rate than women (Shirzad et al., 2024). In England in 2022, men accounted for nearly three-quarters of all drug-related deaths and suicides (ONS, 2023a, 2023c) and two-thirds of alcohol-specific deaths (ONS, 2024a). The existing research on drug, suicide, and alcohol-specific morbidity and mortality as distinct outcomes shows that gender identity influences self-harm behaviours as well as the rate of substance use disorders (Batty et al., 2012; Callanan & Davis, 2012; Meyer et al., 2019). The gender disparity in substance use and suicide deaths has been attributed to certain facets of masculine identity, such as a desire to demonstrate stoicism (Gorski, 2010) and self-reliance (Pirkis et al., 2017) that can encourage substance use, discourage help-seeking behaviour, exacerbate mental ill-health, and promote engagement in high-risk behaviours (Callanan & Davis, 2012; Gough & Novikova, 2020; Seidler et al., 2016). Despite the significance of DoD to public health, what factors help to explain gender differences in rates of these deaths as a single phenomenon remains a critically understudied area of research.

Existing research from the North East of England indicates that the region’s industrial history has played a central role in shaping local constructions of masculinity. Research on working-class masculinities in the region has shown that traditional male identity was closely tied to physical labour, economic provision, and resilience in the face of hardship (McDowell, 2011, 2020; Nayak, 2006). The industrial workplace not only provided a means of financial stability and the ability to serve as the “breadwinner” for one’s family, but also reinforced a sense of masculine worth through skilled, hands-on work and camaraderie with other men (Nayak, 2006; Willis, 2017). However, the collapse of heavy industry and the shift toward a service-based economy has significantly disrupted these identities, leaving many men without the economic and social foundations that once underpinned their sense of self (McDowell, 2011, 2020). Some studies have found that working-class men in deindustrialised communities often struggle to adapt to the kinds of jobs that remain, which are more precarious, lower paid, and frequently require forms of emotional labour that conflict with traditional masculine norms (Nayak, 2006; Nixon, 2006). This dissonance between cultural expectations of masculinity and the realities of the post-industrial labour market has been linked to economic marginalisation, declining self-worth, and an increased risk of harmful coping mechanisms such as substance use and suicide (Gough & Novikova, 2020). However, more recent research in the North East has found that young men specifically generally do not struggle to identify modern jobs that were compatible with traditional masculine values and others were not opposed to work in more feminised forms of employment (e.g. the service sector) (MacDonald & Shildrick, 2018). Understanding how these entrenched masculine norms shape men’s responses to economic and social change is essential to explaining gender disparities in DoD in the North East.

This study explored how stakeholders (professionals whose work related to DoD in Middlesbrough or South Tyneside, but who may not have lived in either community) and community members in two Northern English towns with above national and regional average rates of DoD, Middlesbrough and South Tyneside, understood and explained inequalities in rates of DoD present in their areas. The specific research objectives were:

1. To explore how stakeholders and community members in areas with a high prevalence of DoD understand the causes of DSA morbidity and mortality locally.
2. To learn how stakeholders and community members understand and explain the inequalities in DoD present in their areas.

Findings unrelated to gender-based inequalities in DoD are reported elsewhere (Price, 2024; Price et al., 2024).

## Methods

### Recruitment and Data Collection

Participants were recruited from two local authorities in North East England, Middlesbrough, and South Tyneside, that had above regional and national average rates of DoD in 2021 (ONS, 2023b, 2023c, 2024a). Stakeholders and community members were invited to participate.

Stakeholders were eligible to participate if they worked with residents of Middlesbrough or South Tyneside, and they felt that their work pertained to drugs, suicide/self-harm, alcohol, and/or mental health. The stakeholders recruited worked in a diverse range of professional backgrounds including law enforcement, charity service provision (such as food banks and homeless outreach), mental health treatment, substance abuse recovery support, community organising, local government, public health, and housing management. A number of convenience sampling techniques were used to recruit stakeholders: direct invitations to stakeholders working in a relevant sector, circulation of a study advertisement to a mailing list for stakeholders working in the area, and snowball sampling of stakeholders’ professional networks. 24 stakeholders were recruited for this study. Table 1 provides relevant demographic information.

**Table 1.** Stakeholder Demographic Information

Category	Middlesbrough (number of participants)	South Tyneside (number of participants)
Gender		
Men	6	7
Women	7	4
Age		
18–24	0	1
25–34	1	1
35–44	1	3
45–54	7	4
55–64	4	2
Total participants	13	11

Community members were eligible to participate if they were over the age of 18, able to complete an interview in English, were a resident of Middlesbrough or South Tyneside, and were comfortable discussing DoD. The study was advertised to community members by flyers displayed in public spaces (e.g. libraries, community centres) and in organisations accessed by members of the community (e.g. food banks, welfare-to-work organisations). Participants were also recruited from community groups that were open to the general public (e.g. public living rooms and community drop-ins). 30 community members participated in the study; Table 2 provides relevant demographic information for community members.

**Table 2.** Community Member Demographic Information

Category	Middlesbrough (number of participants)	South Tyneside (number of participants)
Gender		
Men	9	9
Women	7	5
Age		
18–24	1	0
25–34	2	1
35–44	2	0
45–54	3	1
55–64	3	8
65+	5	4
Total participants	16	14

Data were collected through semi-structured interviews and one focus group. Interviews were conducted online using Microsoft Teams, in-person at the participants’ places of work, or in a public setting such as a coffee shop or community centre according to participants’ preferences. One focus group with community members (*n* = 7) was conducted in South Tyneside to accommodate participants’ request to complete their interviews together; the focus group was conducted in a private space at a community organisation. The topic guide (see Supplemental Material) used in interviews and the focus group asked participants for their views on the factors underpinning gender-based inequalities in DoD, as well as how a range of unrelated factors contributed to rates of these deaths.

Participants were provided with a participant information sheet detailing how their information would be used and provided written consent to participate prior to participating in the study. All interviews and the focus group were audio recorded (with consent) and transcribed by the researcher. Identifying information was removed, audio recordings were deleted, and anonymised transcripts were used during analysis. In order to ensure participant privacy, the data used to generate findings are not publicly available and direct quotations used in this publication are not attributed to specific participants. Community members were provided with a £25 supermarket voucher to thank them for participating. Stakeholders were not provided with a voucher as their interviews were completed during their regular working hours.

### Analysis

All participant data were analysed using the Iterative Categorisation (IC) technique developed by Neale (2016), with findings interpreted through thematic analysis (Braun & Clarke, 2006). IC, a method for managing qualitative data analysis, supports rigour and transparency in the analysis process while remaining compatible with other common analytical approaches like thematic analysis. Initially developed to support research on addiction, IC provides a structured framework without being a stand-alone analysis method. Coding was conducted using a deductive coding matrix generated based on the interview topic guide, as the topic guide explored factors, such as socioeconomic status and mental health, that are known to influence DoD risk and health inequalities more generally. As coding progressed, additional codes were generated inductively, allowing for the refinement and merging of codes. Once all data were coded, relevant codes were used to generate themes related to gender-based inequalities in DoD.

The interpretive analysis stage of the IC process seeks to identify patterns, associations, and explanations within the data (Neale, 2021). Interpretive analysis within IC involves three processes: conceptualising, differentiating, and externalising. In this study, conceptualising was undertaken inductively and involved the identification of themes related to gender identity and DoD risk. Data differentiation involves checking themes for similarities, differences, and outliers within participant narratives. Differentiation of participant narratives is conducted based on inclusion in subgroups and characteristics relevant to the study; in this case, participants were differentiated based on gender, area of residence, and their status as a stakeholder or community member. Themes were differentiated to investigate whether participants who expressed similar beliefs shared any discernible characteristics (e.g. if themes present in the narratives of women were different from those in men’s). After differentiation, there were few clear differences between the themes present in participant narratives based on any identifiable characteristics. The similarity between participant narratives regardless of gender indicates widespread agreement among participants and justifies viewing the data provided by both men and women as that of a single group.

### Study Setting

This study was conducted in Middlesbrough and South Tyneside, two deindustrialised local authorities in North East England. While industrial work was central to life in Middlesbrough and South Tyneside from the mid-19th century, both towns experienced rapid and severe deindustrialisation in the latter half of the 20th century (Hudson, 1986, 2005; Telford, 2022; Telford & Lloyd, 2020). Deindustrialisation ushered in widespread unemployment and resulting poverty which persists today; people in both Middlesbrough and South Tyneside experience higher unemployment, and those in work earn lower weekly pay, than both regional and national averages (ONS, 2024b, 2024c). As deindustrialisation took hold in the North East, the work opportunities available to young men rapidly changed, with severe limitations on economic opportunity and a rise in unstable working environments, such as those on temporary contracts or a part-time basis (Nayak, 2006; Telford & Lloyd, 2020). In the economic environment that has arisen after deindustrialisation, low-skill service jobs, such as cleaning, bar work, and retail services, constitute the majority of available jobs in communities like Middlesbrough and South Tyneside, and such jobs are often perceived to be of low social class (McDowell, 2011).

### Researcher Positionality

I am an early career researcher from the United States working in North East England. My main area of reflection during this project was the influence that my experience with gender had on my research. I am a cis-man and present as such, which likely influenced the way people spoke to me about masculine gender roles and how they were experienced by men in these towns. When men spoke to me about aspects of masculine identity, such as the desire for men to be the “breadwinner” of their household or the reluctance of men to use mental health services, they did so with a degree of camaraderie. My gender identity may have also made me more approachable to men and increased men’s willingness to participate in my study. When women spoke to me about masculine gender roles, they often expressed a concern that they might offend me by pointing out what they saw as negative aspects of masculinity. While I tried to mitigate this by reassuring these participants I would not be offended, it is possible that these women would have been more open while speaking to another woman than they were with me. Additionally, this article was written after completion of my PhD thesis, which framed inequalities in DoD as a product of structurally violent political and economic systems; in turn, this will have shaped the lens through which I have analysed and presented these findings.

### Ethical Approval

Stakeholder interviews were granted ethical approval by the Newcastle University Faculty of Medical Science (FMS) Research Ethics Committee (REC) on 01/05/2022 (REF:22812/2022). Ethical approval for community members was received from FMS REC on 23/02/2023 (Ref: 2443/26851).

## Findings

Three themes were generated from participants’ responses surrounding explanations for gender disparities in rates of DoD: Industrial History and Masculinity, Masculinity and Class, and Masculinity as a Barrier to Help-Seeking.

### Industrial History and Masculinity

Participants believed that the culture of deindustrialised places like Middlesbrough and South Tyneside placed significant importance on traditional masculine gender roles and that these helped to explain men’s rates of DoD in their areas. Participants reported that these areas had a “proud heritage of hard work and facing adversity” that men in their areas felt they needed to live up to. Participants connected the strong cultural importance of traditional gender roles to the areas’ industrial legacies.

To answer the question though, I think a lot of this goes back to heavy industry, to mines, to traditional roles. To how men were. Old habits die hard, and people have a view don’t they about how they’re supposed to be as a guy in the North East. – South Tyneside Stakeholder, Man

In Middlesbrough and South Tyneside, it was reported that men believed they should be the breadwinner for their families. Participants said that historically, men in these towns were able to provide for their families by working in industry but felt that this was no longer possible with the kinds of jobs available. Participants felt that men derived their sense of purpose from working and being productive, so when they were not able to do that, it was perceived that men felt like a failure. In turn, this failure to provide for their families was understood to be upsetting and harmful to their self-worth, so they turned to unhealthy coping mechanisms like drugs, alcohol, and self-harm.

I think the things that working-class men would have, a good job to provide for their families, they just don’t have that anymore. I think this sounds, I don’t know like I’m a dinosaur or something. But I think men, men want to work. Everyone wants to work, to be productive. But if the women around you are going off and getting jobs or going to school and you’re left behind and can’t compete, it has a massive effect. – Middlesbrough Community Member, Woman

The socialisation of masculine gender norms, the process by which boys learn the socially expected behaviours and characteristics of men, begins at a young age (Amin et al., 2018; Peate, 2020). Even before a boy reaches working age, “anticipatory socialization,” in which expectations and understanding about work are formed, can begin by observing the working life of men in the boy’s immediate family and surrounding community (Strangleman, 2024). Participants believed that the historical legacy of men working in industry had shaped masculine gender roles in former industrial communities and led to the belief that men should be the breadwinners in their families. Participants in this study suggested that while the available work opportunities had changed from predominantly industrial to predominantly service economy based, the cultural expectations around work for men remained largely the same.

The gender roles have not as progressed as they have in other parts of the UK, here there are still some very backwards views of those sorts of things. I think it’s more accepted here for someone, especially a man, to sit in the pub all day if he’s got nothing else to do, for example. That’s far more accepted here. – South Tyneside Community Member, Man

This theme explains how certain facets of masculine identity in deindustrialised communities, such as self-reliance and the desire to serve as a “breadwinner” combined with the economic opportunities available in post-industrial labour markets, combine to produce DoD for men because existing gendered expectations for work are incompatible with available job opportunities. According to participants, men who were unable to fulfil gendered expectations because of this disconnect experienced low self-worth and used drugs, alcohol, or self-harm as a means of coping or escaping. This finding is relevant not only to Middlesbrough and South Tyneside, but may help to explain why DoD cluster in deindustrialised communities around the world, as sociological research in other deindustrialised areas has demonstrated a similar link between masculine identity and industrial heritage (Rhodes, 2013; Zukin, 1993).

Secondly, we’ve still got that old expectation that men should provide. You were brought up with the “mens should be the provider” type of thing and then if you’re not able to be the provider you lose your self-worth. – Middlesbrough Stakeholder, Man

### Masculinity and Class

It is evident from participants’ narratives that there was a class-based element of the masculine gender roles present in their communities. This is consistent with sociological theories of intersectionality and multiple masculinities, which suggest that social categories such as social class and gender interact to produce systems of (dis)advantage and power (Crenshaw, 1989) and that masculinity is understood differently by different social groups (Connell & Messerschmidt, 2005; Liu et al., 2016). Descriptions of traditionally masculine traits vary significantly across social classes, with upper-class masculinities characterised by qualities such as “confident,” “powerful,” and “ambitious” and working-class masculinities characterised by traits such as “tough,” “hard-working,” and “dedicated” (White & Diekman, 2023). It is notable that in this study participants frequently described men in terms that fit the latter category.

Starting out with being a kid expecting men to be strong, to fight and be tough and try these things and be risk takers. There is that element, the bravado and stereotyping. Then there is the element where they, men, are supposed to work hard, to provide for families and if they don’t, they’re failing. – South Tyneside Stakeholder, Man

The traits commonly associated with working-class masculinity that were considered an asset by industrial employers (Willis, 2017) are now often prohibitive to employment in the service sector (McDowell, 2020). The masculine working-class identity that participants believed was present in Middlesbrough and South Tyneside may be detrimental to men’s ability to enter the post-industrial labour market, and this may help to explain the high unemployment rate and economic instability of men in these areas (and thus, the above average rates of DoD, given that participants believed deaths from these causes were closely tied to men’s failure to serve as “breadwinners”), but it is not the whole story. Working-class men have shown a willingness to reorient their identities through new forms of employment, but often lack the resources necessary to do so, such as education, experience, and job opportunities (Haywood & Mac an Ghaill, 2013; McDowell, 2020; Walker, 2022); this highlights how class-based gender roles intersect with structural determinants such as access to education and job opportunities to produce DoD. It is not just that men in deindustrialised areas like Middlesbrough and South Tyneside are unwilling to work in feminised sectors, it is also that those jobs are difficult to find and offer unstable, low-pay work (Schneider, 2021).

They still have this model that they should be the breadwinner and the proud working man, which was probably the old model of Middlesbrough which is not something that’s there for them anymore. – Middlesbrough Community Member, Woman

The belief that failure to fulfil gendered expectations for work results in distress – leading to negative coping mechanisms – aligns with the concept of masculine gender role discrepancy. Masculine gender role discrepancy occurs when a man perceives himself as failing to meet culturally prescribed standards of masculinity, leading to feelings of inadequacy, shame, and distress (Pleck, 1995; Reidy, Brookmeyer, et al., 2016).

You imagine a man who has supported his family all his life, suddenly he’s out of a job. He’s in an environment where he has no idea how to get on or get back into work. He feels belittled by going to the system and going on the dole. That’s how you’ve lost your self-esteem, your mates from work. You can’t afford to go out the pub on a Friday to see your mates to get back in with them. Can’t find a job. The pressure builds, it’s like a pressure cooker. What would you do? Could you blame him? – South Tyneside Community Member, Man

In this theme, participants described how men in deindustrialised communities were raised with the expectation that they would serve as breadwinners for their families, yet the realities of the post-industrial labour market often made this impossible. This gap between expectation and reality was believed to contribute to low self-worth, frustration, and reliance on self-destructive coping mechanisms such as substance use and self-harm. Existing research has linked masculine gender role discrepancy to increased psychological distress, externalising behaviours, and higher levels of risk-taking, all of which may contribute to the patterns of DoD observed in these communities (Reidy, Berke, et al., 2016; Reidy, Brookmeyer, et al., 2016; Vandello & Bosson, 2013).

So, if you think some of these men, they don’t have jobs, they can’t get a job because they’re addicted to whatever, they’re not fulfilling their purpose to provide. They’re lacking purpose. It’s a recipe for suicide. – Middlesbrough Stakeholder, Man

The class-based element of masculine gender roles outlined in participants’ narratives provides context for understanding gender-based inequalities in DoD and why these deaths disproportionately affect men in economically deprived areas rather than those of higher social status. Men of higher social status were better equipped to navigate the economic changes brought by deindustrialisation, as their identities were less tied to manual labour and more aligned with intellectual and professional achievements (White & Diekman, 2023). In the context of this study, the concept of multiple masculinities provides insight into not just why men die from DoD, but why it is disproportionately men in economically deprived areas (Camacho et al., 2024), highlighting the intersection of class, masculinity, and economic marginalisation as key factors driving these deaths. For DoD research, this underscores the need to account for how class-based gender roles and economic conditions interact to shape vulnerability to despair. It is not that masculine gender roles are solely responsible for men’s rates of DoD; it is the intersection of these gender roles with broader structural forces.

### Masculinity as a Barrier to Help-Seeking

Some facets of normative masculine gender roles were identified as a barrier to accessing mental health services. It was believed that men would not talk about problems with their mental health, self-worth, or their feelings more generally, because to do so was seen as weak and would conflict with the desire to demonstrate stoicism and self-reliance. Participants reported that since men were supposed to be tough, they would choose to conceal their emotions and not seek help when they were suffering. Participants’ belief that these facets of masculine identity were a barrier to men seeking mental health services was a view that has often been voiced in the wider literature (Kupers, 2005; Seidler et al., 2016; Yousaf et al., 2015). Participants believed that peer support opportunities for men were also lacking. Participants reported that men were afraid to open up to their friends, and friends feared being emotionally vulnerable by providing support, which created peer networks in which nobody was able to share emotions without fear of judgement.

Men don’t like to talk about their feelings because that’s a sign of weakness straight away. They have to be the macho man. They have to be seen to be holding it all together and they don’t. They think “Well if I talk to someone I’m less of a man.” I think because they don’t feel like they can open up and talk to people. – Middlesbrough Stakeholder, Woman

Some participants believed that because men were afraid to seek help or share their emotions, they were more likely to engage in negative coping strategies, such as the use of substances, to help them cope with their problems. Participants believed that men preferred external methods of coping with negative emotions – those that allow one to escape from the self – such as drinking and drug use, as opposed to women, who they believed favoured internal coping mechanisms – those that seek to deal with one’s internal problems. There is evidence to support the belief that men are more likely than women to engage in substance use when experiencing a mental health disorder such as anxiety or depression (Cavanagh et al., 2017; McHugh et al., 2018). Participants believed that women were generally able to be more emotionally vulnerable and talk about their feelings, which explained why they were less likely to suffer from DoD.

I would think it’s something to do, especially going down the mental health side of things, as a man, its bloody hard to speak up. As a woman, it’s the norm. Which is good. I think we’re getting to a state in this country where people are encouraging men to speak more, which is good. I would say, what’s hard for men to do is normal for a woman. – Middlesbrough Community Member, Man

Participants felt that men tend to be more violent and aggressive than women, so when men wanted to hurt themselves, they chose more violent means than women did. This belief aligns with existing evidence, which demonstrates that men are more likely than women to use highly lethal methods (e.g. firearm or hanging) when attempting suicide (Barrigon & Cegla-Schvartzman, 2020; Callanan & Davis, 2012). This pattern is part of a broader concept known as the “gender paradox in suicide,” where women report higher levels of suicidal ideation, yet men have a significantly higher rate of suicide completion (Canetto & Sakinofsky, 1998; L. Griffin et al., 2022). There was agreement amongst participants in this study that men’s preference for more violent means of suicide was responsible for the above-average male suicide rate; there is some evidence to support this belief, although research into the gender paradox in suicide has yielded mixed results regarding the underlying causes. Some research has found that the gender paradox in suicide is largely attributable to the methods used by men versus women (Schrijvers et al., 2012; Tsirigotis et al., 2011), while other research has provided evidence that women attempting suicide express less lethal intent than their male counterparts, indicating that suicide may serve different functions for men and women (e.g. intent to escape a situation via death for men, vs. a cry for help from women) (Cibis et al., 2012; Freeman et al., 2017).

With the male population, men don’t like speaking about their feelings and won’t reach out. Statistically, they’re less likely to engage with services and more likely to use more dangerous methods [*of suicide*] and not communicate. Generally, you’re more likely to doing [*sic*] the violent acts that will lead to death more than females would. – South Tyneside Stakeholder, Woman

Participants’ belief in this theme was, in short, that the desire to demonstrate stoicism and pressure to avoid help-seeking due to a desire to embrace self-reliance prevented men from seeking help when they were distressed, and that men used substances or died by suicide as a result. While this belief is consistent with the empirical literature, it neglects important evidence that suggests a more nuanced view of men’s mental health is necessary. When men do overcome social stigma and choose to engage with services, they are often not well served by providers. Clinicians report feeling inadequately prepared to engage with and provide care to men (M. G. Harris et al., 2015; Kingerlee et al., 2014). Men often disengage from treatment early (McKelley & Rochlen, 2010; Swift & Greenberg, 2012) and when they do disengage, they are unlikely to reengage via alternative services (McKelley & Rochlen, 2010; Möller-Leimkühler, 2002). Many men who die by suicide have engaged with a service provider (e.g. mental health provider or general practitioner) in the week before their death (Bachmann, 2018; Chock et al., 2015; Stene-Larsen & Reneflot, 2019). It is therefore reductive to propose that the problem is simply that men do not engage with services; it is also that when they do choose to engage with services, service providers fail to fully engage *them*.

Some researchers have encouraged that we move away from pathological characterisations of masculinity and towards a view that acknowledges the complexities of men’s mental health needs and help-seeking behaviours (Seidler et al., 2018; Stene-Larsen & Reneflot, 2019). Researchers have attempted to learn what clinical approaches are engaging for men and how these approaches can be integrated into a wider range of mental health services. Collaborative client–clinician relationships, as opposed to a standard clinician-led model of mental health care delivery, have been found to be engaging for men (Kivari et al., 2018), and these models are compatible with many evidence-based forms of psychological treatment, such as cognitive behavioural therapy (River, 2018). The online training program *Men in Mind*, developed by men’s health charity, Movember, prepares mental health practitioners to engage and respond to men in therapeutic settings and has shown effectiveness in increasing practitioners self-reported efficacy to work with men, which may improve their efficacy working with male clients (Seidler et al., 2024; Seidler et al., 2022). Outside of traditional clinical settings, informal peer-support groups such as “men’s sheds” have shown effectiveness in improving men’s mental health and have high acceptability with men (Foettinger et al., 2022; Sharp et al., 2022); participants in this study also saw value in peer support groups for men.

You don’t want to come to community groups and sit with a bunch of women who are chittering and knitting. Men are the hardest. You know, I mean, men don’t talk about their feelings and stuff. That’s why I think stuff like men’s groups are so important. It’s a really good thing. Like, look at [*Name*]. He doesn’t look like the kind of guy who’s in touch with his feelings and stuff, but he is. Having him be at the group and talk about his feelings and normalising it is really good. These groups make such a difference. – Middlesbrough Stakeholder, Man

The concept of multiple masculinities proposes that masculinity is not a monolith and that a diverse range of masculine identities develops at the intersection of social determinants of health such as social class, race, and sexuality (Evans et al., 2011), suggesting that to effectively serve men, clinicians and public health practitioners must understand the complex patterns of masculine identity (Seidler et al., 2018; Wenger, 2011). While the evidence surrounding how best to meet men’s mental health needs will continue to develop, it is clear that a more nuanced view of the barriers to effective mental health treatment for men than the one provided by participants in this study is called for.

## Discussion

The findings of this study underscore the need for service providers to adopt a nuanced understanding of masculinity in order to effectively engage men at risk of DoD. Participants consistently reported that some elements of traditional masculine norms, particularly those surrounding stoicism, toughness, and self-reliance, acted as significant barriers to help-seeking behaviours. Participants believed that these traits made men hesitant to access support services and that this reluctance often resulted in men turning to substance use and self-harm as coping mechanisms. To address this, services need to be designed in ways that align with men’s sense of identity, offering support that does not threaten their perception of masculinity. Given the dramatic differences in rates of DoD between men and women (Shirzad et al., 2024), doing so is a critical first step that must be prioritised by service providers. By taking into account the complex relationship between masculine identity, despair, and help-seeking, service providers can make mental health and substance abuse services more accessible and acceptable to men, mitigating some of the gender-based risk factors for DoD. While aligning services with men’s identities offers a promising approach to improving engagement, further research is needed to assess its effectiveness in practice and to ensure that such adaptations lead to meaningful improvements in health outcomes.

Joiner’s Interpersonal Theory of Suicide (ITS) offers a widely recognised framework for understanding suicidality, emphasising perceived burdensomeness, thwarted belongingness, and acquired capability for suicide (Van Orden et al., 2010). These concepts may partially explain aspects of DoD, particularly the distress that participants in this study felt men experience when they were unable to fulfil their traditional “breadwinner” role (perceived burdensomeness) and the impact of economic and social dislocation on social connectedness (thwarted belongingness). Additionally, the normalisation of substance use and self-harm in deindustrialised communities that has been observed in other studies (Blackman, 2010; Price et al., 2024) may contribute to an acquired capability for self-destructive behaviours. However, interpreting the findings solely through an ITS lens would shift the focus toward individual cognitive and psychological factors rather than the broader social, economic, and structural conditions that participants in this study believed shaped these deaths. While ITS helps explain why some individuals may be more vulnerable to suicide and risky behaviours such as substance abuse, it does not fully account for the collective patterns of DoD observed in deindustrialised communities (Camacho et al., 2024; Scutchfield, 2019; Steelesmith et al., 2023). The findings of this study suggest that addressing DoD requires structural interventions – such as economic redevelopment and service design that acknowledges men’s identities – rather than solely individual-level psychological interventions aimed at shifting beliefs.

The themes in this study encourage taking a holistic view of men’s mental health and gender-based inequalities in DoD. Participants believed that normative masculine gender roles in their area placed significant emphasis on the ability of men to serve as the “breadwinner” for their families, but that this was no longer possible in the post-industrial economy; this masculine discrepancy was believed to be distressing and result in moral injury that men coped with through substance abuse and self-injurious behaviours. It is clear from this narrative that influence of facets of normative masculine gender roles on rates of DoD cannot be understood without considering their interaction with the broader political and economic environment. In the years following deindustrialisation, very little meaningful effort was made to stimulate economic growth and promote the development of alternative employment sectors in former industrial areas (Kitson & Michie, 2014; MacKinnon, 2020; McCann, 2016). This lack of investment in economic development coincided with a reduction in welfare benefits and a scaling back of labour rights and collective bargaining power for workers (Albertson & Stepney, 2020; Scott-Samuel et al., 2014).

In combination, the lack of economic development, reduction in welfare spending, and the curtailing of labour rights created an environment in deindustrialised areas in which poverty was common, chances of economic advancement were low, and there was little in the way of a social safety net; these factors were a direct result of the neoliberal economic policies of the Thatcher Government (Hall et al., 1983; Nunn, 2014; Tomlinson, 2021). The normative gender roles that positioned men as the primary breadwinners for their families were, before deindustrialisation, aligned with an economic environment where such a role was realistically attainable through participation in industrial labour. In this context, the cultural expectation that men should be the breadwinner for their household, in and of itself, was not inherently harmful to men. Changes to the labour market and social safety nets in the form of deindustrialisation and government policy took that economic environment away and left men in deindustrialised areas with few realistic opportunities for economic stability or advancement, which, according to participants, left men feeling worthless and at risk of DoD. This suggests that while certain facets of masculinity contribute to DoD risk, the core issue lies in the profound economic changes that have drastically limited acceptable work opportunities for men in deindustrialised regions. As a result, efforts to reduce DoD must go beyond challenging masculine norms and address the broader economic landscape, creating stable, meaningful employment that allows working-class men to regain a sense of purpose and self-worth in the post-industrial economy. While such efforts may not be sufficient to entirely alleviate the burden of DoD in deindustrialised communities, the findings of this study are clear that addressing upstream contributors to distress is critical.

### Global Implications of DoD in Deindustrialised Regions

These findings have significant implications for understanding gender-based inequalities in DoD in deindustrialised areas, both within the United Kingdom and internationally. The intersection of traditional masculine norms with the economic dislocation caused by deindustrialisation suggests that similar patterns are likely unfolding in other areas where industrial collapse has left men unable to fulfil long-established gender roles, such as the U.S. Rust Belt (Allgood et al., 2022; Rotella, 2002). Interventions aimed solely at improving individual mental health or promoting men’s engagement with services are unlikely to succeed without addressing the structural economic inequalities and cultural factors that drive DoD. These findings contribute to a growing understanding of the underpinnings of the rise in DoD and suggest that efforts to mitigate men’s risk of these deaths must account for the complex interplay between economic and gender-based forces that cause them.

### Strengths and Limitations

This study was the first to use qualitative methods to investigate the factors that underpin gender-based inequalities in DoD. The findings are grounded in participants’ lived experiences living and working in areas with above regional and national average rates of DoD. By examining two deindustrialised towns, this study offers a clear and contextually grounded understanding of why DoD disproportionately impact men in this context and may offer insight into the social forces underpinning DoD in deindustrialised regions elsewhere in the world. The stakeholders who participated in this study were from a range of professional backgrounds related to DoD; the diversity of experience present amongst stakeholders enhances this study’s findings and helps to ensure that the conclusions drawn represent the wide range of experiences across these fields.

In interpreting the findings of this study, it is important to consider the sample from which they were derived. The sample used in this study consisted largely of middle-aged and older adults. While there is considerable value in speaking to members of these demographics (as they are the age groups most at risk for DoD), it is likely that younger people may experience and/or perceive masculinity differently than older people. Additionally, it was beyond the scope of this study to explore how other identities (such as race, sexuality, or trans and nonbinary identities) intersect with gender and class to shape experiences with DoD. The relationship between these identities and DoD risk remains understudied and should be an area of focus for future research. This study’s sample included both men and women, meaning that some participants were speaking from their direct experience with masculinity, whereas others were offering outsider perspectives about what men experience. While there was consensus amongst participants and no discernible differences in themes based on participants’ gender, it is possible that speaking to a greater number of participants would have yielded evidence for differences in perspective surrounding the effects of some facets of masculinity. Data were gathered from participants through both interviews and a focus group. Given the semi-public nature of a focus group, it is possible that these seven participants were less open with their beliefs and impressions than participants who took part in an interview. Finally, the findings of this study are not sufficient to establish causation. Factors beyond those identified in this paper likely also contribute to DoD risk for men, and future research will further develop the academic understanding of the relationship between masculinity and DoD risk.

## Conclusion

This study has shown that people living and working in Middlesbrough and South Tyneside felt that gender-based inequalities in DoD arise due to the intersection of working-class masculine identity and the political and economic decisions that have supressed the economic environment in deindustrialised towns. As a result, the findings suggest that it is insufficient to view DoD among men as solely a problem created by masculine gender roles. While it remains vital that we seek to improve access to mental health and substance abuse treatments, design services in such a way that is acceptable to men, and ensure that service providers are prepared to engage men when they present to services, this will be an incomplete solution. Addressing gender-based inequalities in DoD requires reconciliation with the broader political and economic decisions that have made life intolerable for men in deindustrialised places.

## Supplemental Material

sj-docx-1-jmh-10.1177_15579883251329715 – Supplemental material for “They’re Lacking Purpose. It’s a Recipe for Suicide.”: Masculinity and Gender-Based Inequalities in Deaths of Despair in England Supplemental material, sj-docx-1-jmh-10.1177_15579883251329715 for “They’re Lacking Purpose. It’s a Recipe for Suicide.”: Masculinity and Gender-Based Inequalities in Deaths of Despair in England by Timothy Price in American Journal of Men's Health
