# Supplementary material for: “They’re Lacking Purpose. It’s a Recipe for Suicide.”: Masculinity and Gender-Based Inequalities in Deaths of Despair in England
Source: Am J Mens Health. 2025 May 1;19(2):15579883251329715. doi: 10.1177/15579883251329715 (PMC12049619; doi:10.1177/15579883251329715)
Supplement: sj-docx-1-jmh-10.1177_15579883251329715 – Supplemental material for “They’re Lacking Purpose. It’s a Recipe for Suicide.”: Masculinity and Gender-Based Inequalities in Deaths of Despair in England [file sj-docx-1-jmh-10.1177_15579883251329715.docx]

- Tell me a bit about yourself.
- IF Stakeholder: What is your current role? What is an average day of work like for you? Etc.

*TOWN*

- Can you tell me a bit about TOWN and the people who live here?
  - What are some of the biggest challenges you think people in TOWN face?
  - What factors about Middlesbrough have changed over recent years? What impact do you think these have had on the people living here?
  - What are your thoughts on the health and wellbeing of people in TOWN in general? What are your thoughts on mental health in Middlesbrough specifically?
  - Do you think TOWN has a problem with deaths and illness from suicide, alcohol use, and drug abuse? Tell me about that.

*Beliefs about Causation*

- What do you think is driving deaths and illness from suicide, alcohol use, and drug abuse in TOWN?
  - What role do you think mental health plays a role in these problems?
  - What role do you think economic challenges play in these problems?
  - Are there factors that make people more susceptible to these problems? What do you think those are, and what makes you think they’re important?
  - We know from the available data that these problems often disproportionately impact men. Do you have any thoughts on why that may be?
    - Are there any specific groups or demographics, other than men, that you think are particularly impacted by these problems?

*Solutions*

- What is being done in the community to address these issues?
  - What has been done well?
  - What has been unsuccessful?
  - What do you think the major barriers/limitations to addressing these issues in TOWN are?
- Thinking very broadly now— lets imagine you had unlimited resources at your disposal. What changes would you make to solve the problems with drugs, suicide, and alcohol in this community?
